# A novel approach to minimize the false negative COVID-19 diagnosis by inclusion of specific cell markers and multiple sample collection

**DOI:** 10.1016/j.mex.2021.101270

**Published:** 2021-02-13

**Authors:** Amjad Husain

**Affiliations:** a Centre for Science & Society, Indian Institute of Science Education and Research (IISER) Bhopal, MP, India; b Innovation and Incubation Centre for Entrepreneurship, Indian Institute of Science Education and Research (IISER) Bhopal, MP, India

**Keywords:** SARS-CoV-2, COVID-19, RT-PCR, SARS-CoV-2, Human Coronavirus-2, COVID-19, Coronavirus disease 2019, RT-PCR, Reverse Transcriptase PCR, RdRp, RNA dependent RNA polymerase

## Abstract

The SARS-CoV-2 pandemic has caused unpredictable mortality and economic losses globally. With no approved drug for the treatment, the accurate diagnosis of COVID-19 becomes essential. RNA based test takes several hours and require extensive human intervention for RNA extraction and RT-PCR, but it is preferred over the antibody-based detection as the latter does not detect the early stage infections.

The RT-PCR being a gold standard of COVID-19 diagnosis offers highly standardized detection of the SARS-CoV-2 RNA, still vulnerable for false-negative diagnosis due to absence of infected cells in the sample or inaccurate RNA extraction. Hence there is a need to develop alternative protocols and methods for the accurate COVID-19 diagnosis. Here we propose two additional steps in RT-PCR based COVID-19 diagnosis to minimize false-negative detection. The first step involves collection of four samples from an individual. Each sample should be collected from nasopharyngeal and oropharyngeal regions on day 01, mixed together followed by RNA extraction and then repeating the same exercise on day 03. The RNA extracted on day 01 and day 03 must be pooled together to be used in the RT-PCR. Second, we propose the inclusion of the control marker genes specific to nasal goblet cell, type-II pneumocyte and absorptive enterocytes to ensure the specificity of the RNA source. Overall, these additional steps in the proposed method would increase the chances of SARS-CoV-2 detection in the infected population and would limit the false-negative diagnosis of COVID-19 and hence the spread of this disease.

• RT-PCR based COVID-19 diagnosis is vulnerable to the false-negative results due to inaccurate sample isolation or RNA extraction.
• RNA pool of multiple samples from an individual improves the chances of detection of SARS-CoV-2 by RT-PCR.
• Inclusion of specific marker genes would ensure the right RNA source from the desired cell.

## Graphical abstract

**Figure:**
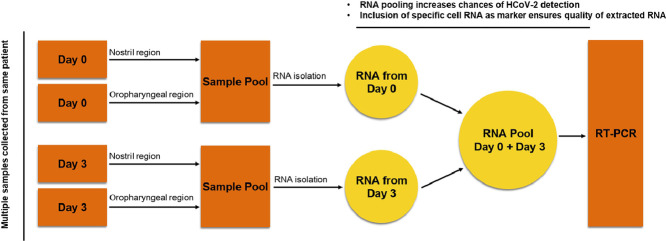

Specification table

**Table:** 

Subject area	Virology, Diagnosis, Molecular Biology
More specific subject area	COVID-19 Diagnosis
Method name	A novel method to minimise the false negative COVID-19 diagnosis by inclusion of specific cell markers and multiple sample collection
Name and reference of original method	Real Time PCR in Virology PMID: 11884626 Improved Molecular Diagnosis of COVID-19 by the Novel, Highly Sensitive and Specific COVID-19-RdRp/Hel Real-Time Reverse Transcription-PCR Assay Validated *In Vitro* and with Clinical Specimens. PMID: 32132196

## Introduction

Severe acute respiratory syndrome coronavirus-2 (SARS-CoV-2) is an enveloped, positive-sense, single-stranded RNA virus that causes coronavirus disease 2019 (COVID-19) in humans. With the relatively larger genome size of around 30 kb compared to the other RNA viruses, the SARS-CoV-2 genome codes for four types of proteins: spike (S), membrane (M), nucleocapsid (N), and envelope (E) [1]. The S protein, being a transmembrane glycoprotein facilitates the virus entry into the host cell. Due to its larger size, it creates distinct spikes on the viral surface. The N protein helps in viral RNA synthesis, while E and M proteins participate in viral assembly. The SARS-CoV-2 belongs to the coronavirus family that contains several other viruses, including MERS-CoV, and SARS-CoV. The SARS-CoV-2 has the ability to spread worldwide through close human interaction or the spilt droplets containing the virus from the infected people. There are possibilities of vertical transmission too, where the virus can be transmitted from parents to the next generation via a placental barrier, during delivery, or through breastfeeding, but vertical transmission in case of COVID-19 was not reported until recently [2]. The first case of vertical transmission of SARS-CoV-2 in India was reported from Sassoon General Hospital, Pune, Maharashtra (India) [3]. The World Health Organization (WHO) declared the COVID-19 outbreak as a pandemic in March 2020. As per the WHO dashboard today on January 21^st^, 2021 there have been 94,963,847 confirmed cases of COVID-19, including 2,050,857 deaths reported to WHO so far (https://covid19.who.int/). An individual with COVID-19 shows symptoms of influenza, such as dry cough, severe headache, fever, and tiredness. In individuals infected with SARS-CoV-2, the critically ill condition may lead to organ function damage, such as acute kidney injury, liver dysfunction, cardiac injury and acute respiratory distress syndrome, which can result in a long-term overall decrease in lung function. Some of the critical cases may also lead to death. The severe conditions and mortalities have been observed more in older patients or patients with a weak immune system [4]. Due to international travel, the SARS-CoV-2 has spread rapidly around the world, causing a pandemic in more than 200 countries, and, the number of infected people and mortalities are on the rise constantly since it got started.

So far there is no new drug developed and approved for the treatment of COVID-19, but there are treatment options adopted worldwide to treat COVID-19 patients. Among those, the convalescent plasma (CP) therapy and drug repurposing offer the potential options of treatment in absence of an approved new drug to treat SARS-CoV-2 infection [5,6].

The convalescent plasma (CP) is the plasma-derived from COVID-19 patients who have successfully overcome the infection. In the past, the CP had been used for the treatment of other deadly diseases caused by viruses such as Severe Acute Respiratory Syndrome (SARS) and Spanish flu, as the plasma provides neutralizing antibodies to the patient against infectious agents. Emil von Behring, a well-known scientist from Germany received the noble prize in 1901 for treating diphtheria using the CP. Although the results of the treatments using the CP have been of mixed types, as there is always a challenge of getting the desirable and right titer of the neutralizing antibody prior to giving the plasma to COVID-19 patients. On the drug development side, there is no new drug developed and approved yet for the treatment, and in such a situation the drug repurposing that uses an existing approved drug for the treatment of another disease may offer the solution [6,7]. It represents an alternative cheaper drug development strategy with a shortened time span of drug development as compared to the traditional new drug development process. Even though the option of using CP has been already in clinical practice, and there are many drug candidates being explored for the repurposing, still a large scale testing of COVID-19 with better accuracy is essential for the epidemiological surveillance, and safely reopening the places after the countrywide lockdowns. Globally, governments are trying to deploy strategies to increase the screening of potentially SARS-CoV-2 infected people. During the initial period of the SARS-CoV-2 outbreak, the infected patients were identified due to the rapid development and widespread use of the reverse transcription-polymerase chain reaction (RT-PCR) method for the diagnosis of COVID-19. Since the virus mutates rapidly being an RNA virus, the sequencing methods are being used to explore novel mutations and the evolution of SARS-CoV-2 isolates [8,9]. These sequencing assays, however, are time-consuming and relatively expensive, which thereby limit their application for the diagnosis of the massive population infected with SARS-CoV-2. Compared to sequencing methods, the RT-PCR based detection offers a cheaper, easier method with a short turnaround time. In addition, the CRISPR-Cas12 based detection of SARS-CoV-2 was also proposed [10], which performs simultaneous reverse transcription and isothermal amplification using loop-mediated amplification (RT-LAMP) for the viral RNA extracted from the patient's samples, followed by Cas12 detection of predefined coronavirus sequences, after which cleavage of a reporter molecule confirms the virus detection. In addition to testing the viral RNA, there are antibody-based tests in practice, which detect specific IgG and IgM in the patient's serum [11,12]. These specific antibodies generated by the immune system against the virus can stay within the blood for a longer period even after the patient's recovery. Therefore, detection of titer of IgG and IgM can provide important information related to the immune response against a specific infection. So far, several commercial immunoassay tests are available in the market for the detection of SARS-CoV-2 antibodies.

Recently, the attempts have been also made using the microfluidic platform for the detection of COVID-19. A microfluidic-based platform allows the diagnosis of a clinical fluid sample via microchannel which offers faster and high sensitivity detection [13]. These tests could be useful as more than one biomarker simultaneously can be detected in a single chip. Lin et al developed the quick multiplexed detection of IgG, IgM antibodies, and antigen of SARS-CoV-2 simultaneously in a single microfluidic chip. They fabricated a microfluidic channel and functionalized it via fluorescent microsphere-labelled capture antibodies [14,15]. The fluid sample flow through the microchannel due to capillary action, and IgG, IgM, and antigen present in the sample bind to the labelled antibodies. Due to this interaction, the fluorescence signal is generated and measured through the detector.

Although the antibody-based tests offer a shorter turnaround time and high specificity, these tests fail to detect the early stage infection in an individual as it takes a few days to get the antibody response after infection. Therefore the RT-PCR test has been the gold standard so far considering the speed, sensitivity and accuracy of the test along with successful detection of early-stage infection. Although, RT-PCR is the most commonly used test for the SARS-CoV-2 diagnosis, there is a huge scope of improving the accuracy and sensitivity of the COVID-19 diagnosis. When inaccurately done, it may lead to two situations. A false-positive diagnosis, which labels a normal individual as SARS-CoV-2 positive or a false negative diagnosis that fails to identify an infected individual. The false-positive individual may undergo unnecessary psychological stress with long quarantine and social isolation; a false negative individual may spread infection around and contribute to the severity of the pandemic [16,17]. The existing single attempt method of sample collection from potentially infected people, and lack of inclusion of required controls in RT-PCR test, makes the process vulnerable to false-negative diagnosis. It is therefore of utmost importance to address this problem since the false-negative diagnosis of COVID-19 leads to further spread of the disease.

## Proposed method

For a diagnostic test to be efficient, the very first step is the collection of samples from the patient. The Centre for Disease Control (CDC) has recommended the collection of upper respiratory specimens, preferably from nasopharyngeal (NP) or oropharyngeal (OP) region for the RT-PCR test. As of June 2020, the CDC has also allowed nasal swabs to be taken by the patient or health worker and used as a valid specimen for testing when NP swabs are not available. The present method of sample collection involves one-time collection from either the NP or OP region of a potentially infected individual. This method does not ensure getting the virus-containing cells or free virus in the samples even if an individual is infected. In the case of low virus titers in NP or OP regions, there are chances of missing out on the infected cells and hence the SARS-CoV-2 RNA. In addition, there may be cases when collected samples have no cells at all, irrespective of an individual's infection status. SARS-CoV-2 can be found in all or any of these specimens of NP, OP and nasal swabs. Testing on each specimen may be necessary depending on the variability of patient condition or a need for re-testing after a negative result, but testing multiple samples from the same individual in RT-PCR would be time taking and expensive. To maximize the chances of getting the virus or infected cells in the samples collected, and to minimize the false negative diagnosis, we propose a 4-swab method. In this method on day-1, the samples must be collected from the NP and OP regions and then pooled together in the Virus Transport Media (VTM) followed by RNA extraction to prepare the RNA pool-A. On day-3, the samples must be collected again from the NP and OP regions, pooled together in the VTM followed by RNA extraction to prepare the RNA pool-B. The RNA pool-A and RNA pool-B must be mixed together, and the mixed RNA can be used as the template source for the RT-PCR. This RNA pool would also contain SARS-CoV-2 RNA if the individual to whom the samples belong, is infected. The pooling of samples from NP and OP regions and then mixing the RNA samples from day-1 and day-3 would enhance the chances of SARS-CoV-2 RNA detection, even if the sampling method has missed the virus at one of the two sites or attempts. There has been evidence that the chances of detection of the virus from an infected person are 66% in the case of nasal samples, and 33% in the case of pharyngeal samples. Considering these events as mutually exclusive, the probabilities of detection would be higher when more than one sample is collected from NP and OP regions and pooled together. Even though this method would require sample collection twice from the same patient, it can be easily implemented on the patients admitted in hospitals, or people undergoing quarantine. Also, using the pooled RNA as template source would bring down the frequency of false-negative diagnosis.

Once the RNA is isolated and pooled, the presence of SARS-CoV-2 RNA can be tested using the RT-PCR. In RT-PCR, the SARS-CoV-2 RNA, if present in the RNA pool is reverse transcribed to make the complementary DNA (cDNA), which is then amplified into numerous copies using specific primers. Different regions of viral RNA such as E gene and RNA dependent RNA polymerase (RdRp) can be amplified with specific primers in real-time to confirm the presence of SARS-CoV-2 RNA in the patient samples.

To avoid the false-negative diagnosis, different kits have included specific internal controls such as external RNA added at the time of RNA extraction and then tested during the RT-PCR to ensure that the negative diagnosis is not due to RNA degradation. Also, multiple kits use RNAse-P as an internal control to ensure the stability of RNA as well as the source of the RNA isolated, but it is not universally included in all the diagnostic kits, currently being used for the COVID-19 diagnosis. Also, RNAse-P confirms the presence of cellular RNA but since it is expressed in multiple cell types, it does not qualify to be a signature marker of a specific cell potentially infected with the SARS-CoV-2. Therefore, the inclusion of cell-specific controls becomes significant to minimize false negative COVID-19 diagnosis in RT-PCR based methods.

To confirm the presence of cells in the samples collected, ideally, the sample should contain the RNA from the goblet secretory cells of the nasal passages, type-II pneumocyte of lungs and absorptive enterocytes of the intestine, the cell types which are known to get infected by SARS-CoV-2. At least one out of these 3 genes should get amplified in RT-PCR irrespective of the presence of SARS-CoV-2 RNA in that sample or infection status of that individual. No amplification of even 1 out of these 3 genes would suggest the absence of those specific cells in the sample or inaccurate RNA extraction. Samples showing amplification of any of these 3 genes and no amplification of viral genes should be considered as true negative. Even after the inclusion of these genes as internal controls, there is still a possibility that an individual being infected is not releasing enough of the virus into NP or OP regions to be picked up and detected, but the chances would be minimal due to multiple samples and using the RNA pool for the RT-PCR test as described earlier.

**Fig. 1:**
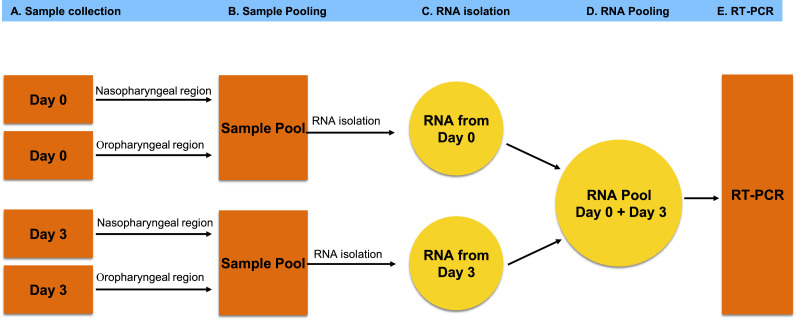
**Overview of multiple swab method of sample collection from potential COVID-19 patients.** The RNA pool prepared from multiple samples (isolated from NP and OP regions on Day-1 and Day-3) of same patients should be used in RT-PCR along with inclusion of specific cell markers as internal controls to minimize the false negative diagnosis of SARS-CoV-2 infected indivuduals.

Using pooled RNA for RT-PCR, and inclusion of specific marker genes from goblet nasal secretory cells, type-II pneumocytes of lungs and absorptive enterocytes of the intestine will not only minimize the frequency of false-negative detection, it would also help in limiting the spread of the virus by identifying and containing such patients in the long run. Overall by reducing the false-negative diagnosis of COVID-19 significantly, this method will contribute to limit the further spread of the current SARS-CoV-2 pandemic Fig. 1.

## Declaration of Competing Interest

Author has no conflict of interest in regards to the information shared in the manuscript.
